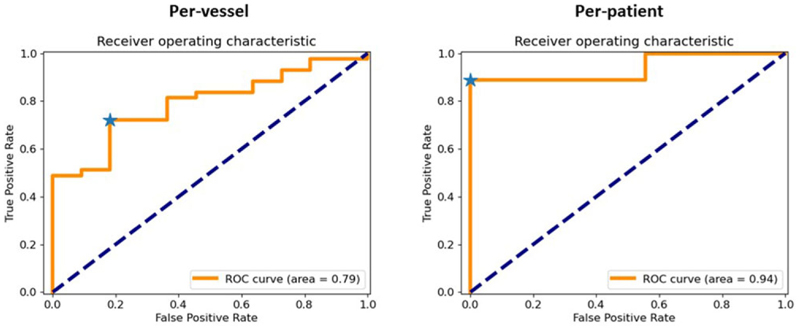# Automated quantitative myocardial perfusion cardiac magnetic resonance with artificial intelligence-based arterial input function correction versus fractional flow reserve

**DOI:** 10.1093/ehjci/jead119.397

**Published:** 2023-06-19

**Authors:** CM Scannell, R Crawley, NCK Wong, E Alskaf, A Chiribiri

**Affiliations:** Eindhoven University of Technology, Eindhoven, Netherlands (The) King's College London, London, United Kingdom of Great Britain & Northern Ireland

## Abstract

**Background:**

Quantitative myocardial perfusion cardiac magnetic resonance (QP-CMR) is gaining importance in clinical practice due to the increasing availability of automated processing software. However, a significant obstacle in the widespread deployment of QP-CMR is the accurate estimation of the arterial input function (AIF), as this is precluded by the non-linear relationship between observed MR signal and the concentration of contrast agent.

Modern solutions favour a dual-saturation acquisition sequence, acquiring specific low-resolution images with a short saturation time to minimise the signal saturation of the AIF. However, this approach is limited by the availability of the pulse sequence. Recently, the AI-AIF has been proposed (1), which uses an artificial intelligence (AI)-based correction of the AIF, without the need for a dual-sequence or dual-bolus acquisition.

**Purpose:**

In this work, we compare QP-CMR values, quantified with the AI-AIF versus the invasive reference standard: fractional flow reserve (FFR), to identify diagnostically significant coronary artery disease (CAD), as defined by FFR < 0.80.

**Methods:**

This study enrolled 18 patients (mean age 65 (± 11), 8 female) who underwent stress perfusion CMR (Philips Achieva 3T) and invasive FFR assessment of at least one coronary artery. Perfusion was quantified in a fully-automated manner with motion compensation (2) and AI-based segmentation and processing (3), using a Fermi function-constrained deconvolution. Fully automated pixelwise perfusion maps were generated, and further subdivided into 16 myocardial segments. Segments were assigned to their respective perfusion territory (4) and the QP value for a territory is given as the mean of its two lowest segments. Receiver operating characteristic (ROC) analysis was performed to identify cut-off thresholds and report the diagnostic accuracy of the proposed approach.

**Results:**

The overall mean QP-CMR value was 2.29 ml/min/g (± 0.32). Figure 1 shows QP maps and FFR values for 3 example patients. The mean QP-CMR value was 2.09 ml/min/g (± 0.36) in patients with significant CAD and 2.95 ml/min/g (± 0.48) in patients without significant CAD. In total, 28 vessels underwent FFR assessment with 12 vessels in 10 patients being positive for significant CAD (FFR < 0.80). The mean QP-CMR value in FFR-positive coronary territories was 1.76 ml/min/g (± 0.37). The ROC curves are shown in Figure 2 on a per-vessel (left) and per-patient (right) level. The optimal QP threshold on a per-vessel level is 2.11 ml/min/g and on a per-patient level is 1.96 ml/min/g.

**Conclusion:**

QP-CMR is feasible with the AI-AIF and does not require dual-sequence acquisition or a dual contrast injection. The QP values derived after the AI-based correction of the AIF show good agreement with FFR assessment of epicardial coronary disease. However, due to the limited sample size, further work is required to confirm these findings.

## Figures and Tables

**Figure:**
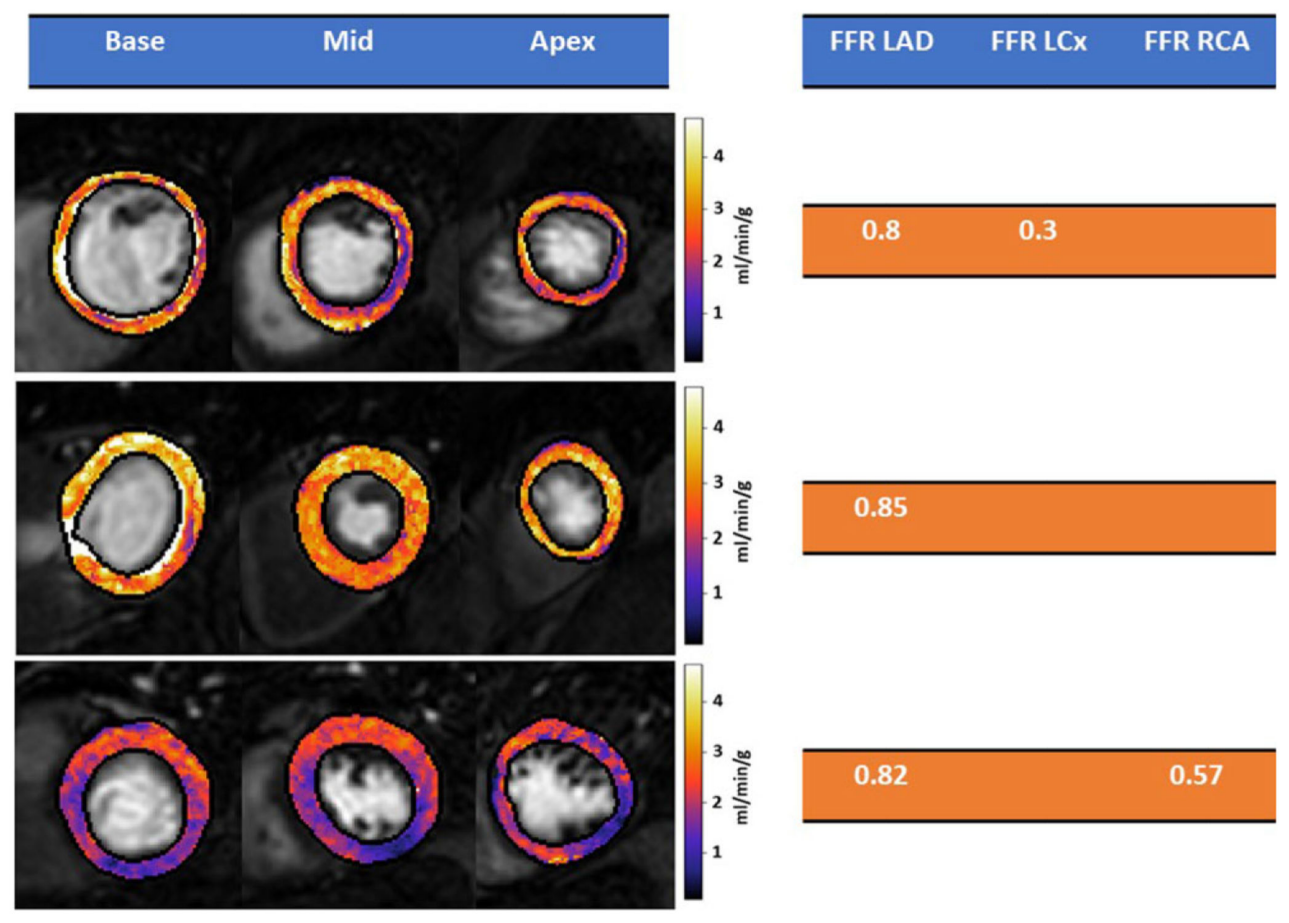


**Figure:**